# Self-Unity as Ground Zero of Learning and Development

**DOI:** 10.3389/fpsyg.2019.00414

**Published:** 2019-03-28

**Authors:** Philippe Rochat

**Affiliations:** Department of Psychology, Emory University, Atlanta, GA, United States

**Keywords:** self, self-unity, development, infancy, early cognition, self-awareness

## Abstract

Contrary to the suggestion that we are born in a state of confusion and primordial state of a-dualism with the environment, infancy research of the past 40 years shows that from the outset, infants are objective perceivers guided by rich evolved survival values of approach and avoidance in relation to specific resources in the environment such as faces, food, or smell. This starting-state competence drives and organizes their behavior. Evidence-based ascription of self-unity at birth is discussed. Selected findings are presented suggesting that self-unity is a primordial human experience, the main organizer of behavior from the outset. Self-unity is the necessary ground zero enabling the rapid learning and development taking place early in human life.

Are we born disorganized and in need of building an awareness of the self as an organized entity among other entities? Or, on the contrary, are we born with experiential self-unity and awareness that with maturation and experience become conceptual? Much progress in infancy research of the past four decades suggests that the latter is most likely and in particular that without an initial sense of self-unity, infants would and could not develop the way they do.

Self-unity as the embodied sense of self as an *organized and differentiated entity among other entities* is ground zero of learning and development. This is true for both empirical and common sense reasons. Without such experiential unity at the origins of development, it is difficult to conceive how consciousness in general might develop, and in particular, how self-consciousness could develop the way it is described by current child studies, emerging from around 18 months of life with social emotions like embarrassment or shame (Rochat, 2009).

The driving argument here is that learning and development early in life and beyond would rest on a primordial and necessary sense of self-unity. The question is not anymore whether such experiential unity exists from the get-go, but rather what it is made of and how it manifests itself early in the life of the individual. This, I would assume, could represent important grounding information for designers of complex artificial learning systems trying to mimic human children in their rapid and rather canalized development as this article tries to show.

## Ascribing Experiential Self-Unity at Birth

Immanuel Kant over three centuries ago already proposed that the sense of an embodied unity is a primordial foundation of being phenomenally conscious about something. According to Kant in his critique of Pure Reason (Kant, 1781/2007), impinging sensations from the world, including sensations from the own body as an entity among other entities in the world, become synthesized into patterns of representations eventually forming higher concepts (Brook, 1994). Current infancy research demonstrates that infants from birth do manifest unity in a Kantian sense. In particular, research show that from birth on, infants are responsive to more than discrete, isolated sensations. From birth, they differentiate sensations that originate either from within or without the body (Rochat, 2011).

Infants are born objective perceivers and actors, not simply reflex machines (Rochat and Senders, 1991; Rochat, 2001). An abundance of recent empirical evidence calls for radical revisions of strong-held beliefs and premises from which highly influential theories were built. Newborns display much more than reflexes (Piaget, 1936), a-dualism (James, 1890), or blind auto-eroticism and primary narcissism (Freud, 1905/2000). From the get-go, they behave as differentiated and organized embodied entities among other entities. We are not born in a primordial state of un-differentiation or confusion with the environment (see Rochat, 2011 for further discussion).

It appears that newborns are not just bombarded by meaningless sensory stimulations. If that were the case, we would expect newborns’ behavior to be fundamentally disoriented, a mere collection of responses that would jerk them around in a disorganized manner. Ample evidence demonstrates that this is not the case (Rochat, 2001). Newborns learn and actively explore their environment, even showing evidence that pre-natal experience and learning are transferred into post-natal life (Prechtl, 1984; Hepper, 2002; Hata et al., 2010).

In recent years, researchers have established striking evidence demonstrating, for example, that few hour old newborns show active preference in hearing their mother’s voice compared to another female voice (DeCasper and Fifer, 1980), or that they tend to orient toward the scent of their mother’s amniotic fluid experienced in the womb compared to the scent of the amniotic fluid of a female stranger (Marlier et al., 1998a,b). Newborns transfer prenatal experience and learning into postnatal life. They memorize and recall procedural knowledge over time, orienting head and mouth significantly more when, for example, the stimulation is food or any events associated with food and comfort (faces, posture, or certain tastes as well as smells, e.g., Marlier et al., 1998a).

In short, newborns’ behavior shows plasticity and is not limited to the here and now of random stimulation but includes systematic self-exploration. Van der Meer and Lee (1995), as a case in point, demonstrated, for example, that neonates engage in systematic exploration of their own arms and hands when plunged in the dark with just a thin beam of light cutting across their visual field. These findings, among many others (see Rochat, 2001), point to an experiential awareness from the outset that is organized within a stable spatial and temporal organization.

## Body Schema at Birth

In relation to the body as a whole, hand-mouth coordination systematically associated with the engagement of the feeding system, as in this case of the drop of sucrose on the tongue of the infant (Blass et al., 1989), is in itself suggestive that newborns do possess rudiments of a body schema (Gallagher and Meltzoff, 1996, see also Butterworth, 1992 for a similar argument). This primitive body schema is not rigid, changing and being re-calibrated as a function of rapid motor and postural progress in the weeks following birth (e.g., developing use of hands to reach, grasp, and explore objects). The organized behavior expressed in hand-mouth coordination implies some mapping of the body whereby regions and parts of the own body are actively and systematically (as opposed to just randomly) put in contact with each other, in this case hands and mouth with a straight and orchestrated spatiotemporal trajectory. Hand-mouth coordination is also well documented in fetuses. Already during the last trimester of gestation (Hata et al., 2010), hands and mouth move in an organized and coordinated fashion, following predictable spatiotemporal patterns with signs of motor anticipation (i.e., mouth opening in anticipation of manual contact with the mouth, without any visual guidance, see also Butterworth and Hopkins, 1988).

More recent observations vindicate the existence of a body schema at birth and in the first week of life. Filippetti et al. (2013) observe that healthy newborns aged between 12 and 100 h presented visually with a pair of faces of another infant stroked with a brush and prefer to look at the child’s face touched in perfect synchrony with strokes applied by an Experimenter on their own cheek. Most striking is the fact that this significant preference vanishes when the two faces of the other infant being stroked are inverted by 180 degrees (i.e., upside down presentation). These findings demonstrate that newborns detect multisensory (i.e., visual-tactile) synchrony, but to the extent that it is related to their own body schema (canonical right side up face orientation). These observations show that infants from birth do engage in body perception guided by a canonical spatial representation of the own body, i.e., a body schema (Filippetti et al., 2013).

Other data using novel experimental paradigms further support the idea of early body perception, particularly evidence of an interoceptive sensitivity. Maister et al. (2017) observe that 5-month-old infants prefer to look at an animated character that moves on a screen out of synchrony with their own heartbeat, when presented side by side with another character moving in exact synchrony with their own heartbeat. Interestingly, infants who demonstrated the strongest visual preference were also those showing brain (EEG) signals that correspond to the heart evoked potential typically reported in adult studies. Maister et al. also report that infant’s interoceptive sensitivity is particularly salient when infants are presented with animated characters displaying negative emotions, which presumably increases their autonomous cardiac response.

Meltzoff et al. (2018) report new electroencephalographic data collected on 60-day-old infants demonstrating that the neural representations of tactile stimulations applied on different parts of the infant’s body are topographically analogous to the well-documented somatosensory cortex organization of adults. These data further support the idea of an organized body schema from the outset of development, or at least early in the first week following birth (i.e., 2 months, Meltzoff et al., 2018). Finally, although remaining controversial, evidence of neonatal imitation would be another expression of an implicit body awareness and representation (body schema) whereby the sight of active bodily regions in another person (the model) is mapped onto homologous regions of the own body (Meltzoff and Moore, 1977).

In all, body schema and the active propensity of neonates to bring sense modalities and regions of their own body in relation to each other are now well documented. This, in itself, supports the idea that infants sense their own body from birth as an invariant spatial structure, as rudimentary and in need of further refinement. This structure is obviously not Euclidian in the sense of not synthesized (represented) in the mind of the young infant as a precise map of accurate spatial coordinates and configurations. It does not yet entail that the infant has already a re-cognizable image of her own body (a body image). This structure is essentially topological in the sense that it is made of focal attractor regions on the body surface that have great degrees of freedom and a high concentration of sensory receptors such as mouth and fingers. This topology is embodied in action systems that are functional from birth and drive early behavior.

## Implicit Self-Awareness in Neonates

Evidence of a body schema at birth provides some theoretical ground for the ascription of implicit self-awareness from the outset (Rochat, 2009, 2011). Neonates behave in relation to their own body in ways that are different, when compared to how they behave in relation to other physical bodies that exist in independence of their own (Lee and Aronson, 1974; Butterworth and Hicks, 1977; Jouen and Gapenne, 1995). They feel and demonstrate from birth a distinct sensitivity to their own bodily movements *via* proprioception and internal (vestibular) receptors in the inner ears. New data also demonstrate that newborn perception can be modulated by a sensitivity to their own heartbeat (Maister et al., 2017). Interoceptive, proprioceptive, and vestibular sensitivities are well developed and operational at birth. They are sense modalities of the self par excellence.

As expression of self-world discrimination, neonates root significantly more with head and mouth toward a tactile stimulation from someone else’s finger than from their own hand touching their cheek (Rochat and Hespos, 1997). Rather than being in a state of fusion and confusion with the environment, few hours old infants pick up visual information that specifies movements of their own body or ego-motion while they in fact remain stationary. Like adults sitting in a stationary train while watching another train moving, neonates experience the illusion of moving. Research demonstrate that, like us, they adjust their bodily posture according to changes in direction of an optical flow that is presented in the periphery of their visual field (Jouen and Gapenne, 1995). This kind of observations point to the fact that from birth, infants are endowed with the perceptual, qua inter-modal capacity to pick up and process *self-specifying* information (Butterworth, 1992; Rochat, 2001).

Neonates experience the body as an invariant locus of pleasure and pain, with a particular topography of hedonic attractors, the mouth region being the most powerful of all, as noted by Freud years ago. Within hours after birth, in relation to this topography, infants learn and memorize sensory events that are associated with pleasure and novelty: they selectively orient to odors associated with the pleasure of feeding and they show basic discrimination of what can be expected from familiar events that unfold over time and that are situated in a space that is embodied, structured within a body schema. But if it is legitimate to posit an a-priori “embodied” spatial and temporal organization of self-experience at birth, what might be the content of this experience aside from pleasure, pain, and the excitement of novelty?

The proprioceptive sense of the body is, from birth on, a necessary correlate of most sensory experiences of the world. As proposed by Gibson (1979), to perceive the world is to *co-perceive* oneself in this world. In this process, proprioception or the muscular and skeletal sense of the body in reference to *itself* is indeed the sense modality of the self. From birth, proprioception alone or in conjunction with other sense modalities specify the own body as a differentiated, situated, and eventually also agent entity among other entities in the world. This corresponds to what Neisser (1988, 1991) first coined as the “ecological self,” a self that can be ascribed to infants from birth. As pointed by Neisser (1995), criteria for the ascription of an ecological self rests on the behavioral expression by the individual of both an awareness of the environment in terms of a lay out with particular affordances for action and an awareness of the own body as a motivated agent to explore, detect, and use these affordances (Neisser, 1995; Rochat, 2011).

Newborns appear to meet the criteria for such awareness. They also seem to possess an a-priori awareness that their own body is a distinct entity that is bounded and substantial, as opposed to disorganized and “airy” (Rochat, 2001, 2011, 2012). Immediately after birth, infants perform self-oriented acts by systematically bringing hand to mouth, as already mentioned. In these acts, the mouth tends to open in anticipation of manual contact and the insertion of fingers into the oral cavity for chewing and sucking (Blass et al., 1989; Watson, 1995; Rochat, 2011). What is instantiated in such systematic acts is what would amount to an *organized body schema* (Rochat, 2012). These acts are not just random and cannot be reduced to reflex arcs. Hand and mouth are coordinated and not automatically triggered. It is a systematically orchestrated activity oriented toward an oral goal. It constitutes an open-looped and flexible system in contradistinction to the basic constitution of reflexes that are triggered and automatic, fundamentally closed-loop systems.

Hand-mouth coordination in neonates needs to be construed as functionally self-oriented acts proper. Because they bring body parts in direct relation to one another, as in the case of hand-mouth coordination, they provide neonates with invariant sensory information specifying the own body’s quality as bounded substance, with an inside and an outside, specified by particular texture, solidity, temperature, elasticity, taste, and smell.

As discussed in previous works on the origins of self-perception and self-consciousness (Rochat, 2011, 2012), the a-priori awareness of the own body as a bounded substantial entity is evident in neonates’ postural reaction and gestures when – for example – experiencing the impending collision with a looming visual object, an event that carries potentially life-threatening information. In a classic study performed years ago, it was reported that neonates aged 2–11 weeks manifest head withdrawal and avoidant behavior when exposed to the explosive expansion of an optic array that specifies the impending collision of an object. When viewing expanding shadows specifying an object either receding or on a miss path in relation to them, infants do not seem not manifest any signs of upset or avoidant behavior (Ball and Tronick, 1971). In a follow-up experiment, Carroll and Gibson (1981) report that 3-month olds facing a looming object with a large aperture do not show signs of avoidant behavior. Rather, they are reported leaning forward as if they wanted to look through the aperture. These observations indicate that very early on infants manifest what seems to be an a-priori awareness of their own body as substantial: a unified entity among other entities occupying space, thus potential obstacles and source of collisions.

## Conclusion: Self-Unity and Development

I tried to show that behavioral research over the past 40 years and current studies based on novel physiological and behavioral recording techniques (Maister et al., 2017; Meltzoff et al., 2018) demonstrate that the human neonate has rudiments of an experiential self-awareness that has unity, this unity justifying ascription of an implicit and embodied self-unity at birth, in other words the ascription of a minimal and necessary self-awareness from the outset of development.

In relation to development, the question is not how we eventually become mindful and self-aware from a starting state of confusion. It is not how we eventually become endowed with a strong mind pulling out of a primitive state of computational weakness, non-differentiation, and selflessness. Rather, based on what we now know about neonates, the question is how does the implicit awareness of the embodied self expressed already at birth come to be also explicit and conceptual by the second year, as children become self-conscious proper. How the experiential *I* eventually becomes the conceptual *Me*, and what might drive such development?

That is the perennial question of developmental psychology that not only infant and child researchers but also evolutionary and comparative psychologists keep tackling on all fronts (see Rochat, 2018). This effort is based on a new generation of behavioral paradigms trying to capture self-consciousness in human ontogeny, using, for example, as proxies first physiological signs of embarrassment (Lewis et al., 1989), and the sense of being potentially evaluated by others (emergence of evaluative audience perception – EAP, see Botto and Rochat, 2018).

In recent years, developmental cognitive neuroscience research yielded new neural markers of experiential awareness at birth, and even during the fetal stages of development. For example, first evidence of consciousness might be correlated with the development of functional neural pathways that link thalamus and sensory cortex already by the third trimester of gestation, or even earlier with the emergence of functional pathways necessarily involved in conscious pain perception (Lee et al., 2005). If there is a renewed effort in mapping pre- and post-natal brain growth, using neural markers that would correlate with levels of consciousness achieved by children in their development, we are still far from explaining the actual mechanisms that would drive such development. If there is a positive correlation between brain growth and levels of consciousness, including levels of embodied self-consciousness achieved by the child (see Zelazo et al., 2007), we are still far from a causal explanation.

The argument of unity and selfhood at birth rests on the idea that the development of self-awareness, from the implicit *I* to the conceptual *Me*, presupposes a representation to begin with what Zelazo (2004) labels “minimal consciousness” in his model of consciousness development. It is this minimal “embodied” consciousness in the newborn that I tried to emphasize in this article. However, aside from the empirically informed depiction of a starting state awareness and the distinction between various levels of experiential awareness and representation expressed by children in their development, the question of what might be the causes or developmental triggers of processes such as the spontaneous representational re-description mechanism proposed some years ago by Annette Karmiloff-Smith (1992) remains wide open. This is particularly true in light of the fact that such process appears to exist prior to language which is often considered as the major determinant of reflexive consciousness and meta-cognitive capacities, what Vygotsky (1978) viewed as internalized thinking derived from language acquisition.

Language and its progressive mastery do certainly play a causal role in the development of new explicit levels of consciousness. We do not have to assume that language shapes the mind, to recognize that language use by the child in interaction with scaffolding others and its progressive mastery does unquestionably contribute to children’s reaching new levels of abstraction and representational re-description. But to a large extent, we are still very much agnostic as to what might trigger such re-description prior to language and what might lead infants in particular to re-describe their starting-state unity and sense of selfhood to eventually become explicit and conceptual about it. We can assume, however, that from the outset, social interactions with more advanced and linguistically competent others play a central role in infants’ advances toward more abstract levels of embodied self-awareness (Vygotsky, 1978; Tomasello, 2019).

These developmental issues form a challenge that is worth embracing because the way children develop and what develops in their experience of the world, including their own body can reveal much of the building blocks and layers of human consciousness in general, human self-consciousness in particular (Rochat, 2003).

Those designing and building learning machines could gain from evidence regarding the self-unifying starting state of newborns, the “ground zero” of rapid learning and development in infancy and beyond.

## Author’S Note

This article is a novel elaboration and synthesis of similar ideas developed previously (e.g., Rochat, 2001, 2011, 2012, 2016, see reference list).

## Author Contributions

The author confirms being the sole contributor of this work and has approved it for publication.

### Conflict of Interest Statement

The author declares that the research was conducted in the absence of any commercial or financial relationships that could be construed as a potential conflict of interest.
